# Validation of a computational model of bone conduction sound reception in mysticetes

**DOI:** 10.1371/journal.pone.0334393

**Published:** 2025-11-14

**Authors:** Petr Krysl, Margaret A. Morris, John A. Hildebrand, Ted W. Cranford

**Affiliations:** 1 Department of Structural Engineering, University of California, San Diego, California, United States of America; 2 Scripps Institution of Oceanography, University of California, San Diego, California, United States of America; 3 Department of Biology, San Diego State University, San Diego, California, United States of America; Natural History Museum of London, UNITED KINGDOM OF GREAT BRITAIN AND NORTHERN IRELAND

## Abstract

Computational models serve as useful complements to physical experiments, but they require validation to build confidence in their applicability. This study outlines the validation of biomechanical models for mysticete sound reception, specifically using experiments involving an instrumented gray whale skull exposed to underwater sound. Detailed descriptions of the models are provided. The models were evaluated using a set of similarity metrics applied to both measured and computed frequency response functions. While high-quality agreement was not achieved, the models corresponded reasonably well with observed experimental data. A sensitivity analysis examined the models’ responses to variations in input material properties. Although these changes in material properties influenced model response, they accounted for only modest changes in similarity. A more significant challenge to achieving higher accuracy was the mismatch between the acoustic waves generated in experiments and the models’ assumption of plane wave loading. Despite this, the models successfully captured important biomechanical behavior, such as the enhancement of motion of the tympanic bullae relative to the basicranium. Model validation remains an ongoing endeavor, and this study represents an initial step.

## 1 Introduction

Knowledge of the low frequency sound-reception capabilities of mysticetes is needed to assess the potential impact of anthropogenic sound on their anatomy and behavior [1]. This is increasingly important due to the presence of noise from shipping traffic, seismic exploration, and offshore development in whale habitats [2]. In particular, knowing the frequency sensitivity of hearing for a variety of whale species would provide insight into their vulnerability to anthropogenic disturbance.

Studies of mysticete hearing are rare due to the difficulty of conducting behavioral or electrophysiological studies. This is due to their immense size and the difficulties associated with holding them in captivity. As a result, these animals must be studied by indirect methods, for instance with behavioral observations [3].

An alternative approach is to numerically model the hearing apparatus of whales, estimating sound reception and sound exposure without the need to access live animals [4]. Computational models have consequently become attractive alternative strategies [5]. Several advantages accrue from the use of such models: (i) live specimens are not required, and thus the practical and ethical issues associated with animal husbandry can be avoided; (ii) in contrast to experiments *in vivo*, models allow testing of multiple scenarios in the same specimen or model; (iii) features of the model may be adapted at little cost; (iv) estimating mechanical quantities such as velocities and pressures at internal anatomic locations may be inaccessible in specimens but visible and accessible in models; (v) studying the influence of one specific feature, such as the input parameters or the geometry is enabled, and the effects of uncertainty can be quantified. Perhaps most importantly, (vi) the fundamental mechanisms of sound transmission can be studied holistically, since the well-known obstacles of physical experimentation (limited access, not enough specimens, limited capabilities of sensing) are impediments no longer. A modeling approach also enables studies of the effect of exposure to specific frequencies without the extra noise and confounding variables that may be present in experiments on live animals These advantages are illustrated for instance in our prior work, which demonstrates the utility of modeling for investigating mysticete auditory processes [4,6–15]. We also acknowledge the limitations of modeling compared to experiments. In experiments, the physical mechanisms appear without any assumptions or idealizations, with their full richness and complexity.

### 1.1 Mysticete bone conduction hypothesis

The tympanoperiotic complex (TPC) plays a crucial role in whale hearing. This functional unit is composed of three bony structures: the periotic contains the cochlea and is firmly embedded into the base of the skull; the tympanic bulla is suspended from the periotic by two flexible pedicles; and the ossicular chain, fused to the bulla at the malleus, that connects the incus to the stapes footplate at the oval window of the inner ear [16]. An anatomic representation of the TPC in the gray whale can be found in Fig 1 of Morris et al. [17].

**Fig 1:**
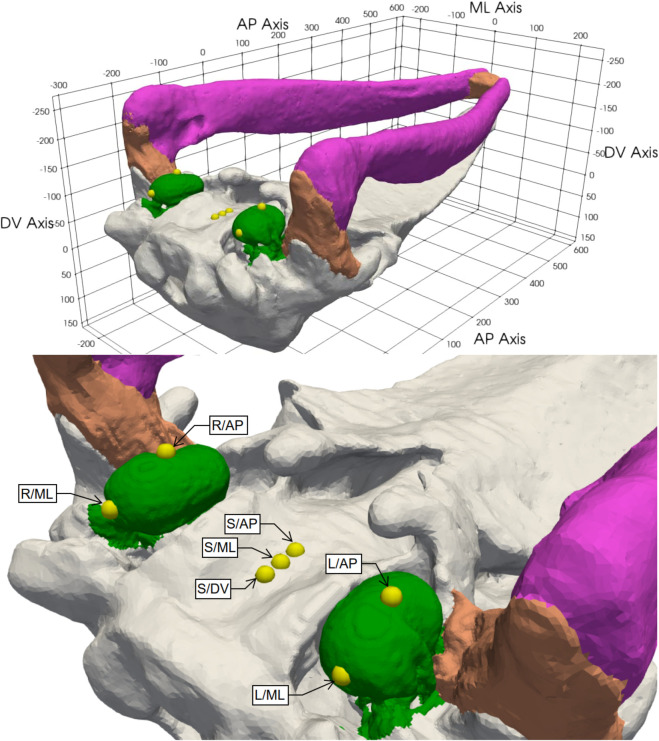
Natural skull: Posterolateral view of the inverted skull. Cranium – light gray, lower mandible – magenta, temporomandibular joint and mandibular symphysis – orange, TPC – green. Sensor locations shown as yellow spheres. AP = anteroposterior axis, ML = mediolateral axis, DV = dorsoventral axis.

Mysticete hearing is facilitated by the conventional mammalian mechanism: the ossicular chain is set in motion, and the stapes footplate consequently drives fluid movement within the cochlea [14]. In mysticetes, the ossicular chain mechanically links the tympanic bulla to the periotic bone, which houses the inner ear. Therefore, the most plausible mechanism for setting the ossicles in motion is a vibration of the bulla relative to the periotic bone [18].

A key question is: Which mechanisms generate the relative motion between the tympanic bulla and the periotic bone? Two possible mechanisms were proposed by Cranford and Krysl [4]:

1. Sound waves could propagate through the animal’s soft tissues, pushing on the bony surfaces of the TPC, such that sound pressure on the bulla may generate a force, causing the bulla to vibrate relative to the periotic;
2. Sound waves impinging on the animal’s head excite vibrations of the bones comprising the skull, and the bullae suspended on flexible pedicles respond with amplified resonant motion relative to the periotic.

The second mechanism can be described as a form of bone conduction. Not in the narrow sense implied by the use of this term in investigations of human hearing, but in the general sense encompassing any pathway through which waterborne acoustic energy is transmitted to the inner ear via cranial bone involvement [19].

The computer simulations of Cranford and Krysl [4] demonstrated that pressure focusing (mechanism 1 above) was largely ineffective in the whale anatomy at frequencies whose wavelengths in water were much longer than the dimensions of the spaces surrounding the TPC. For example, the wavelength of 1 kHz sound waves is approximately 1.5 m in seawater, and the bulla is roughly of the size of a human fist. Based on their modeling results, Cranford and Krysl [4] hypothesized that bone conduction is the predominant hearing mechanism for such low frequencies. The hypothesis has now been validated with physical experiments [18].

### 1.2 Validation of a model of bone-conduction sound reception

A prerequisite for using computational models to understand mysticete sound reception is validation (a critical step in any field that relies on numerical simulation [20]). It is helpful to distinguish between verification and validation: the former is “solving the equations right” (verification), whereas the latter is “solving the right equations” (validation). Validation assesses whether the numerical model accurately represents the physical reality. The two Vs often appear in conjunction with uncertainty quantification, and the whole process is then known by the acronym VVUQ (Verification, Validation, and Uncertainty Quantification).

Morris et al. [17] reported on experiments designed to investigate sound-induced vibration of the gray whale skull (*Eschrichtius robustus*). Both a denuded natural juvenile gray whale skull and its 3D printed plastic replica were submerged and exposed to sound waves generated by acoustic transducers. The accelerations of the tympanic bullae compared to the basicranium were measured. In addition, the natural juvenile gray whale skull, the plastic replica and an adult gray whale skull were also tested in air, with excitation supplied by a Unholtz-Dickie model 5PM mechanical shaker [17]. Overall, these measurements span a broad range of anatomical configurations and excitation conditions, offering a robust dataset for testing computational models of mysticete bone-conduction hearing.

The objective of this paper is to report on validation of a computational model designed to predict the motion characteristics of the gray whale skull when exposed to underwater sounds under conditions equivalent to those of the experiments. If the model successfully captures the dynamic features observed in the experimental data—such as relative motion between the bullae and the skull and frequency dependent response characteristics, the model may be considered validated.

### 1.3 Significance

The high mass density of the bony ear complexes and their firmly embedded attachments to the skull are conserved anatomical features across the Mysticeti, suggesting that the underlying mechanisms of sound reception are similar among all baleen whales. Interactions between incident sound waves and the skull produce deformations that drive motions in each bony ear complex, resulting in heightened sensitivity for low-frequency sounds. This predominantly low-frequency sensitivity has significant implications for assessing the impact of mysticete exposure to anthropogenic sounds. Anthropogenic noise in the ocean has increased steadily over the past half century. The results presented here bolster the argument that computational models can provide a valuable tool for regulatory agencies and large-scale industrial users concerned with minimizing impact on the ocean environment .

### 1.4 Outline

The structure for the paper is as follows. First, the physical artifacts used in the experimental components of the study are introduced. Then the two computational models used in the study are explained: the first model simulates a pressure field to which the specimens are exposed underwater, and that pressure field is then used as a boundary condition for the second model which predicts the dynamic response of the test specimens. The second model allows for high-fidelity, efficient sweeps through frequency to compute frequency response functions relating the input pressure loading to accelerations of selected locations on the tested specimens. The results are then presented with the use of similarity metrics applied to the frequency response functions. Finally, the discussion highlights the sources of uncertainty. In particular, we discuss the influence of material parameter uncertainty, and the effects of uncertainties associated with the underwater acoustic field of the physical experiments.

## 2 Materials and methods

The validation experiments used in this study are documented in detail by Morris et al. [17]. In the present work, we use the underwater experiments performed on a natural juvenile gray whale skull and its 3D printed plastic replica. Both specimens were instrumented with accelerometers, as shown in Fig 1, producing frequency response functions of acceleration per unit pressure ($m/s^{2}/Pa$). While Morris et al. [17] reported these measurements as velocity transfer functions (in units of m/s/Pa), in this study we compare with the original measured accelerations. As in Morris et al. [17], we use smoothed recordings of the accelerations to remove some artifacts caused by the finite size of the pool.

### 2.1 Gray whale skull: Natural and plastic replica

#### 2.1.1 Natural skull.

For the physical experiments, the natural juvenile gray whale skull was prepared by the removal of soft tissues superficial to the skull. Although some soft tissues remained within cavities during the experiments, we consider their acoustic properties to be equivalent to water, and hence they are not part of the computational models presented here.

#### 2.1.2 Plastic replica skull.

The plastic replica of the skull was fabricated at a 1:1 geometrical scale using ASA (acrylonitrile styrene acrylate) thermoplastic of grade F900 [21]. The skull bones and interconnecting ligaments were segmented as a single object from the CT scan data and rendered in the same plastic. Due to the size constraints of the 3D printer, the replica skull was printed in three parts and then glued together. In the final stage of preparation, the surface was finished (smoothed) using Bondo and painted white. Care was taken not to change the stiffness of the printed part by applying only a very thin layer of the finish.

### 2.2 Models

We next describe the two computational models, PRESS and HVA, that together constitute a modeling pipeline used to produce frequency response curves for comparison with the experiments. It is important to emphasize that both the natural skull and the plastic replica have their own respective pair of models. While the geometries are identical, the material properties and hence the vibrational responses are different. For the sake of brevity, we will only describe the pair of models developed for the natural skull.

#### 2.2.1 Model of the acoustic pressure loading (PRESS).

The acoustic pressure distribution on the surface of the skull when submerged in the TRANSDEC pool was not directly measured. Therefore, we employed a computational model to estimate the pressure loading. This estimated pressure field was then used as input to the second model which evaluated the response of the skull to such loading.

The pressure-distribution model, referred to here as PRESS, was implemented using the vibroacoustic toolkit, VATk, a combination of bespoke FEM software [6], anatomic geometry obtained from X-ray CT scans and mechanical measurements of tissue properties. The equation of motion in discrete form was integrated in time using the centered difference algorithm [6]. This modeling framework has previously been used to study transmit beam formation and sound reception in Cuvier’s beaked whale, *Ziphius cavirostris* [22]. PRESS has also been validated using a physical experiment of sound transmission in a bottlenose dolphin cadaver [11,23].

**Fig 2:**
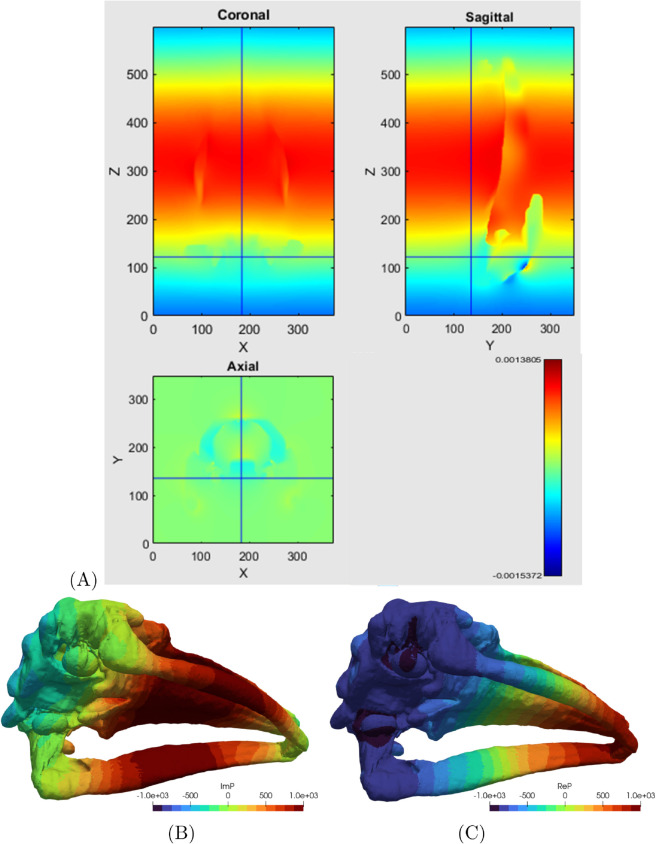
Acoustic pressure loading. (A): Sample VATk PRESS model result – The imaginary component of the total pressure for 1000 Hz incident wave as a volumetric field, visualized on slices (i.e. two-dimensional cuts) through the volume. The volumetric field (A) transferred to the surface of Mesh 3 as acoustic pressure loading for 1000 Hz. The imaginary (B) and real (C) component; pressure magnitude in MPa.

The PRESS model was used to predict the total acoustic pressure distribution around the skull immersed in an infinite extent of water. The geometry of the specimen was represented by a three dimensional array (volumetric dataset) consisting of 373×348×598 voxels, with voxel dimensions of 1.9504×1.9501×1.954 mm. The model simulates the time evolution of the acoustic field by integrating the equations of motion forward in time for the duration over several periods of the incident plane-wave, where the number of periods is chosen to yield an approximation of a steady-state train of waves. The pressure distribution was recorded at eleven time stations during the final period of oscillation, and the resulting sequence of 3D pressure fields was converted into a complex representation with a pair of images, representing the real and imaginary component of a harmonic wave interacting with the scatterer. As an example of the imaginary component of the total pressure at 1000 Hz is shown in Fig 2.

Although the VATk model is capable of computing accelerations of the various bones with sufficient resolution for direct comparison between the model and experiment at any particular frequency, the model is expensive to run. Up to several days of uninterrupted computing is needed per frequency. Conducting a sweep in the frequency domain with as many frequencies as there were in the physical experiments (416) would have been exceedingly expensive. Therefore, given the experimental range of frequencies, 170 to 1000 Hz, the PRESS model computed the pressure distribution for a discrete set of frequencies at intervals of 100 Hz. To reconstruct the pressure distribution at any intermediate frequency, interpolation in frequency was employed. The resulting volumetric pressure fields were then converted to surface pressure fields to be applied as boundary conditions for the second-stage of modeling. The effect of the interpolation is to trade off additional error for much increased efficiency. This error is quantified in Sect 4.3.

#### 2.2.2 Model of the harmonic vibration response of the skull (HVA).

This section describes the second stage of the modeling pipeline, the Harmonic Vibration Analysis (HVA), which computes the skull’s dynamic response to acoustic pressure loading using finite element methods. The computational procedures described here were implemented in the Julia programming language [24,25] in the framework of the FinEtools.jl Julia package [26]. The computational domain of the skull was subjected to known pressure as the boundary condition on all the wetted surfaces of the skull and other tissues.

We adopted the finite element formulation described in [27], which is based on nodally-integrated four-node (first order) tetrahedra with stabilization. Unphysical (spurious) vibration modes of the nodally integrated elements are removed through energy-sampling stabilization. This formulation has been shown to be coercive and locking-free by numerical inf-sup tests, making it suitable for modeling nearly-incompressible materials such as soft tissues. The two stabilization parameters introduced in [27] have been adjusted to *a* = 2 and *b* = 3 to increase the fixed-mesh accuracy. The aim of this modification was to take advantage of the nearly equilateral tetrahedral elements typical of the meshes used in acoustics simulations.

The tetrahedral meshes were derived from the CT volumetric image of the gray whale head. Each voxel in the dataset was classified as either empty space or one of four materials: cranium bone, temporomandibular joint and symphysis ligaments, mandible, and tympanoperiotic complex (TPC). The tissues of the head were tiled with tetrahedra by subdividing each non-empty voxel into five tetrahedra. This initial mesh with approximately 19 million tetrahedra was subsequently coarsened under constraints that enforced relatively fine mesh in the vicinity of the TPC and relatively coarse mesh elsewhere [28]. The meshes were concurrently smoothed using the Taubin volume-preserving algorithm [26].

The finite element model discrete equations result from a weighted residual equation, as described in detail in [27], yielding the matrix expression of the damped harmonic forced vibration

$$\left[-\omega^{2}𝐌+\sum_{m}\left(1+i\eta_{m}\right){𝐊}_{m}\right]\hat{𝐔}=\hat{𝐋}\;,$$ (1)

where **M** is the mass matrix and ${𝐊}_{m}$ is the stiffness matrix of the *m*-th material, $\eta_{m}$ is the loss factor of the *m*-th material [29], and the vector of the nodal loads $\hat{𝐋}$ expresses the effect of the acoustic pressure $\hat{p}$ on the “wet” boundary.

Eq 1 can be solved efficiently by employing a modal decomposition. The necessary number of mode shapes was rather high for the natural skull, 400, most likely due to the fact that the relatively bulky bundles of flexible connective tissue had many natural modes below the limit of 1.5×1000=1500 Hz intended to ensure a tight approximation power of the reduced modal model.

The boundary conditions at the interface between the skull and the surrounding water consisted of prescribed acoustic pressures. These pressures were derived from the output of the PRESS model and applied as loading in the HVA model. Fig 2 illustrates both the imaginary and real components of the total acoustic pressure within the water surrounding the skull and also within the tissues of the skull. The pressure on the surface of the skull in the HVA model was derived by re-interpolating the pressure from the three dimensional images computed by the model PRESS. A comparison between the image of the imaginary component of the pressure on the surface of the bones with the volumetric image of that pressure is shown in Fig 2.

**Fig 3:**
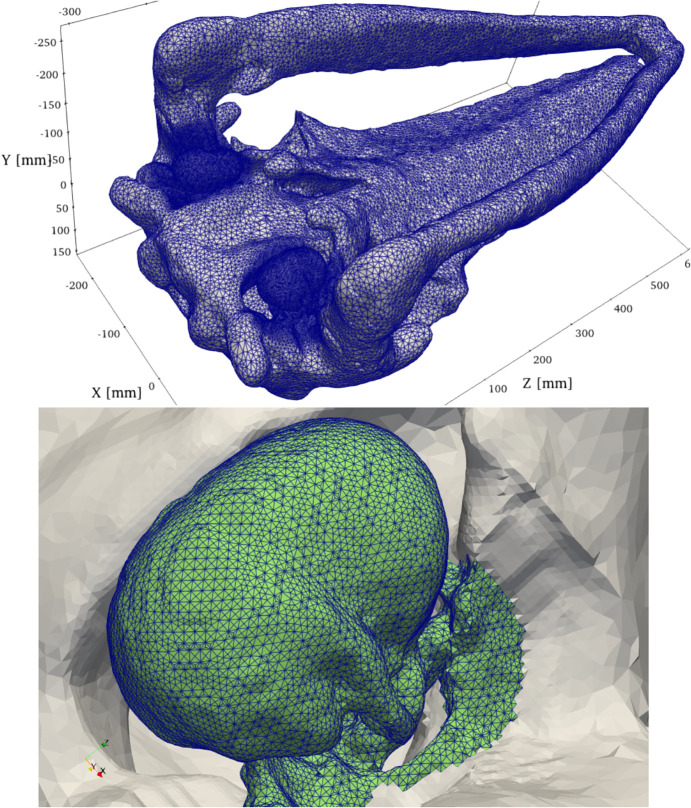
Mesh of the skull. Mesh 6. Element edge length: smallest approximately 2 mm, largest 12 mm. The mesh is refined around the tympanoperiotic complexes to increase accuracy while maintaining simulation costs at reasonable levels. On the bottom: Element edge length on the tympanoperiotic complex is approximately 2 mm. The display of the temporomandibular joint and the mandible was suppressed for clarity.

Fig 3 shows the geometry of the natural skull on the mesh number 6 (see Sect 3.2). Element edges are not shown, as they would not be distinguishable from each other at this resolution, making the figure unintelligible. The cranium and the mandible are connected by the connective tissue of the temporomandibular joint and the left and right ramus of the mandible are joined at the mandibular symphysis. The bones of the bulla, the ossicles, and the pedicles are considered separately from the major bones of the skull.

In the underwater experiments, the skulls were exposed to sound waves generated by a low-frequency transducer, with acoustic pressure as the input. The dynamic response was quantified by accelerations on the tympanic bulla and on the basicranium. They were measured at seven distinct locations with uniaxial accelerometers (Fig 1; also see Fig 3 of [17]). The same sensors and locations were employed for both the natural skull and the plastic replica.

#### Suspension.

During the physical experiments the skull was suspended in the pool in a fixture [17]. The skull was contained within a large-mesh monofilament net bag, which was itself suspended from two horizontal metal pipes which were fastened to a vertical pole. To counteract buoyancy, weights were used to keep the skull and net submerged. For the plastic skull, a 5 lb (approximately 2.3 kg) weight was attached to the net, for the natural skull a 10 lb (approximately 4.5 kg) weight was suspended from the rostrum and a 5 lb weight was wrapped around the mandibles. The pre-stress in the net and in the cords on which the weights were suspended generated a stiffness in the horizontal directions (i.e. the coronal plane). Together with the mass of the skull this gives us an estimate of the natural frequency of the skull suspended in the fixture of approximately 0.4 Hz. However, the frequency range of interest here was between 170 Hz and 1000 Hz, two to three orders of magnitude higher then the natural frequency of the suspended skull. Therefore, the inertial force in this range dominated the resisting force of the fixture by at least four orders of magnitude, and consequently the stiffness of the fixture was neglected.

### 2.3 Material parameters

#### 2.3.1 Natural skull.

A brief discussion of the selection of the material parameters is presented below. Table 1 lists the best-estimate properties of the natural skull used in this paper.

**Table 1:** Material properties. Natural whale skull: Nominal values of the mass density *ρ*, Young’s modulus *E*, Poisson ratio ν, and the loss factor of isotropic damping *η.*

Material	ρ $\left[kg{𝐦}^{-3}\right]$	*E* [GPa]	ν [ND]	η [ND]
Skull, mandible	1100	8	0.3	0.1
TPC	2500	35	0.3	0.01
TMJ, symphysis	1100	0.2	0.49	0.1

*Elastic modulus.* The elastic properties of the bones of the cranium and the mandible are undocumented, and an educated guess is required. Experimental data on the mechanical properties of whale bones are exceedingly rare. One of the few available studies, by Tsukrov et al. measured the stiffness of a right whale mandible [30]. Matching the deflection of the mandible in bending allowed them to deduce the Young’s modulus of the cortical bone as ≈8 GPa. The Young’s modulus of the trabecular bone was expected to be significantly lower, due to its highly porous character. A trabecular bone can also be expected to possess a reduced mass density compared to the cortical bone.

Since the gray whale bones in our study were from a young animal, they were likely softer (i.e. less mineralized and therefore more flexible) than those of an adult, and the Young’s modulus of ≈8 GPa reported by [30] may well represent an upper bound for the cortical bone in the present specimen.

The mechanical properties of the bones of the TPC have been studied in the literature due to their unique properties: they are highly mineralized, making them exceptionally stiff and brittle [31]. Currey studied the bulla of a fin whale [32] and estimated the Young’s modulus to be 20 GPa. Lees et al. studied the ossicles of the fin whale [31], and measured density and the speed of sound. Using the appropriate formula for the conversion of the speed of sound to an elastic modulus in solids, they found results consistent with the [32] estimate for the bulla. The elastic modulus of the ossicles of mysticete whales were determined by Tubelli et al. to be ≈35 GPa [33], the value also adopted by [34].

The mechanical properties of the temporomandibular joint (TMJ) in mysticetes are completely unknown. Given that the joint is subject to extreme mechanical loading while the animal feeds, it is probably quite stiff. In this study, we adopt 0.2 GPa as a reasonable estimate of the Young’s modulus. For simplicity, we neglect the considerable complications associated with the highly anisotropic character of this fibrous tissue.

*Poisson ratio.* Poisson’s ratio relates the transverse strain of a sample to an imposed axial strain. Values for Poisson’s ratio range from 0 to 0.5. Here we adopt 0.3 for all bones. For the connective tissue, which is water-saturated and hence nearly incompressible, we adopted 0.49 as an approximation. The influence of the uncertainty of the Poisson ratio on model predictions has been studied and was found to be insignificant [34].

*Mass density.* The mass density of the TPC has been reported to be approximately $2500\;kg.m^{-3}$ [31]. In contrast, the mass density of trabecular bone of the right whale was determined to be approximately $1100\;kg.m^{-3}$ [30].

*Damping.* Damping behavior in bone remains an area of considerable uncertainty. Lim et al. [35] adopt the loss factor of cortical bone in the acoustic regime as 0.3. (The lack of sources for this number in [35] – none were cited – is telling.) Lakes’ book proposes loss factors lower than this, but the condition and preparation of the bone plays a large role [36]. [37] concluded from experiments aiming to determine the viscoelasticity of human bones that the high-rate loss factor was likely to be around 0.05. Here we presume that the skull has a loss factor of 0.3, but for the TPC we adopt 0.05.

#### 2.3.2 Plastic replica skull.

The material properties used in the models of the plastic gray whale skull are discussed below.

*Elastic modulus.* The replica skull was built by fused deposition modelling (FDM), a process known to introduce anisotropy into the elastic properties of the final structure due to the printing process. However, the detailed orientation and pattern of the deposition is unknown. To simplify the modeling, the material was therefore treated as isotropic, and the elastic modulus was taken to be the mean of the values provided for the two orientations, “on edge” and “upright”, i.e. (2.05+2.14)/2=2.095 GPa as reported in the ASA datasheet [21].

*Poisson ratio.* The Poisson ratio was not reported in the manufacturer’s datasheet [21]. In the absence of specific data, we adopt a typical value of 0.3 as an albeit arbitrary approximation.

*Mass density.* Mass density of the ASA plastic material was provided in the manufacturer’s datasheet as $1080\;kg.m^{-3}$ [21]. However, a more accurate value was derived by weighing the artifact and using the known volume to yield $1008\;kg.m^{-3}$. This probably reflects some residual porosity in the FDM-printed solid.

*Damping.* A frequency-independent loss factor (loss tangent) of tanδ=0.05, was used to estimate material damping in the ASA plastic material, consistent with reported measurements at room temperature [38].

#### 2.3.3 Properties of the water environment.

In all models, the mass density and speed of sound of the water environment were assumed to be: $\rho_{w}=1026\;kgm^{-3}$, $c_{w}=1507\;ms^{-1}$. This is based on properties of the temperature and salinity of the TRANSDEC pool.

### 2.4 Sensors

Instrumentation of the skull with acceleration sensors is shown in Fig 1 and also in Fig 3 of [17]. Two types of uniaxial sensors were used to measured the vibrations on the tympanic bulla. Anteroposterior motion was measured using PCB 333B50 accelerometers positioned on the ventral keel of the bulla. The mediolateral motion was measured with ASC 4221MF-002 accelerometers, which were positioned on the posterior apex of the bulla.

In our simulations, the sensors were accounted for by adding mass to their attachment points on the tympanic bulla. The mass of the ASC 4221MF-002 was 3 grams, while the mass of the PCB 333B50 was 7.5 g. The mass of the attached wires was accounted for by taking 0.25 m of each lead at 12 g/m. Note that the moving mass of the bulla was approximately 250 g. The sensors on the skull were ignored, as their mass compared to the mass of the skull was negligible.

## 3 Results

### 3.1 Metrics

Both experimental and computer modeling simulation results are represented with frequency response functions (FRF), which describe system behavior in the frequency domain. To quantitatively compare FRFs, we employ scaled inner products that range from 0.0 (no correlation) to 1.0 (perfect correlation). An overview of such metrics is provided in [39] .

In this work, we use three FRF metrics: the Cross Signature Assurance Criteria (CSAC) [40], the Cross Signature Scale Factor (CSSF2) [41], and the FRF Similarity Measure (FRFSM) [39].

1. CSAC– Cross Signature Assurance Criterion. CSAC measures shape correlation between the two FRFs. Because the shape of an FRF is largely determined by the location and width of its resonance peaks, this metric is sensitive to the distribution of mass and stiffness within the system [40].
2. CSSF2 - Cross Signature Scale Factor. CSSF2 measures differences in amplitude between two FRFs and hence takes into consideration damping. We use the variant developed by [41], to eliminate the dependence on the CSAC.
3. FRFSM - FRF Similarity Measure. FRFSM is defined using the probability density function of the normal distribution in the frequency domain for two FRFs on a decibel scale [39]. This metric quantifies overall similarity across the frequency spectrum.

Each metric is applied to each of the measurements for the seven virtual sensors in turn. To estimate the variability of the similarity metric *s* (where *s* is one of CSAC, CSSF2, and FRFSM) across the sensors, we use the robust statistical approach based on the median absolute deviation (MAD) from the median [42]. First, the median similarity metric is computed across all seven sensors:

$$\tilde{s}={median}_{j=1,\ldots ,7}\left(s_{j}\right)$$ (2)

where *s*_*j*_ is the value of the metric for sensor *j*. Subsequently, the MAD is evaluated from

$${\tilde{s}}^{mad}=b\times {median}_{j=1,\ldots ,7}\left(\left|s_{j}-\tilde{s}\right|\right)$$ (3)

Here *b* is needed to make the estimator consistent for the parameter of interest. In the case of the parameter with a normal distribution, we set *b* = 1.4826 [43,44].

### 3.2 Discretization error

Control of the discretization error was managed by employing a sequence of progressively refined finite element meshes. Each step involved approximately a doubling of the number of nodes and elements. The coarsest mesh (13) had 63,229 nodes, and then three more meshes (10, 6, and 3) were used with 115,850, 236,612, and 475,989 nodes, respectively. Fig 3 illustrates the discrete HVA model with mesh 6, calling attention to the refinement around the tympanoperiotic complexes.

A detailed view of the tympanoperiotic complex as represented by mesh 6, with approximately 1.2M tetrahedral elements, is also shown in Fig 3. The edges of the tetrahedra are comparable to the dimension of the voxels in the 3D image that served as the basis of the PRESS model.

Table 2 presents the FRF similarity metrics for the replica skull using the refinement sequence of meshes 13, 10, 6, and 3 as described above. The similarity metrics quantify the differences between the FRF curves obtained on successively refined meshes. The results demonstrate satisfactory convergence behavior across all three metrics, and the results on mesh 3 can be considered to have negligible discretization error.

**Table 2:** Convergence of the HVA model for the replica skull. Similarity measures between the FRFs at the seven virtual sensors obtained with HVA models using meshes refined from *m* to *n* (indicated as m→n). Similarity reported with the median and the confidence interval in parentheses as ($\tilde{s}$, $2{\tilde{s}}^{mad}$).

Mesh refinement	CSSF2	CSAC	FRFSM
13 → 10	(0.990, 0.002)	(0.966, 0.015)	(0.946, 0.011)
10 → 6	(0.988, 0.015)	(0.949, 0.054)	(0.940, 0.030)
6 → 3	(0.999, 0.000)	(0.992, 0.006)	(0.988, 0.010)

### 3.3 Underwater measurements with the replica skull

The agreement between the experimental and modeled FRFs is shown in Table 3. The similarity metrics are computed as a composite across all seven sensors. Table 3 presents the median of the metric together with the associated confidence interval. Among the metrics, the CSSF2 tends to have higher scores and may be considered more “optimistic” than the others (in the sense that the metric indicates a closer agreement). In general, the metrics indicate some agreement between the experiment and the model, but far from perfect (i.e. a score of 1.0). To illustrate the variability in agreement, the FRFSM metric has the following similarity measures for the individual sensors: S/AP: 0.767, L/AP: 0.412, R/AP: 0.420, S/ML: 0.255, L/ML: 0.258, R/ML: 0.315, and S/DV: 0.540.

**Table 3:** Comparison with experiments for the replica skull. Similarity measures between the FRFs at the seven virtual sensors obtained with HVA models using mesh 3 and the experiments. Similarity across the seven sensors reported with the median and the confidence interval in parentheses as ($\tilde{s}$, $2{\tilde{s}}^{mad}$).

Experiment date	CSSF2	CSAC	FRFSM
28Aug2023	(0.630, 0.192)	(0.567, 0.245)	(0.412, 0.190)
19Oct2023	(0.807, 0.284)	(0.374, 0.180)	(0.374, 0.158)

**Fig 4:**
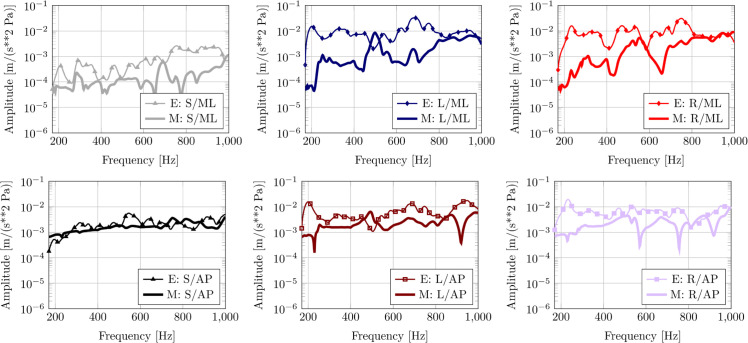
Experiment 28Aug2023 with the replica. Experiment FRF (E) compared with HVA FRF computed with model (M). Key: S = skull; L = left bulla; R = right bulla; ML = mediolateral direction; AP = anteroposterior direction. Top row: Motion of skull and the left and right tympanic bone in the mediolateral (ML) direction. Bottom row: Motion of skull and the left and right tympanic bone in the anteroposterior (AP) direction.

Fig 4 shows the frequency response function (FRF) curves for the mediolateral motion for experiment 28Aug2023 conducted on the plastic replica skull. The mediolateral (ML) response is of particular interest as the swinging motion of the bullae in this direction is likely translated readily into an action of the ossicles (see [18] for a detailed discussion and animations). In general, the model tends to underpredict the ML accelerations of the tympanic bullae in the lower frequency range, up to roughly 400-500 Hz.

Furthermore, Fig 4 shows the frequency response curves for the anteroposterior motion. These results reveal that the amplification of the motion at the keel of the tympanic bulla is underestimated by the model compared with the experiment.

An additional experiment was performed with the plastic replica skull (19Oct2023). The difference between the two experiments on the plastic replica skull, 28Aug2023 vs. 19Oct2023, can be quantified by comparing the two rows in Table 3: the computational model is the same between the two tests, but the experimental results are slightly different. Table 4 shows the similarity metrics for the two experimental datasets. We could consider this to be indicative of the unavoidable experimental error associated with physical testing the plastic replica. The testing of the natural skull would introduce analogous experimental error, probably of equal or larger magnitude. This could not be quantified, as we only had collected one dataset from the natural skull.

**Table 4:** Comparison of two experiments for the replica skull. Similarity measures between the FRFs at the seven virtual sensors measured for two experiments with the replica skull. Similarity across the seven sensors reported with the median and the confidence interval in parentheses as ($\tilde{s}$, $2{\tilde{s}}^{mad}$).

Comparison of	CSSF2	CSAC	FRFSM
28Aug2023 vs. 19Oct2023	(0.972, 0.039)	(0.793, 0.064)	(0.791, 0.040)

### 3.4 Underwater measurements with the natural skull

The agreement of the experimental and computed FRFs is reported in Table 5. For the FRFSM metric the following similarity measures have been calculated for the individual sensors: S/AP: 0.256, L/AP: 0.326, R/AP: 0.243, S/ML: 0.529, L/ML: 0.548, R/ML: 0.289, and S/DV: 0.591.

**Table 5:** Comparison with experiments for the natural skull. Similarity measures between the FRFs at the seven virtual sensors obtained with HVA models using mesh 3 and the experiments. Similarity across the seven sensors reported with the median and the confidence interval in parentheses as ($\tilde{s}$, $2{\tilde{s}}^{mad}$).

Experiment date	CSSF2	CSAC	FRFSM
13Sept2023	(0.898, 0.062)	(0.282, 0.045)	(0.326, 0.120)

**Fig 5:**
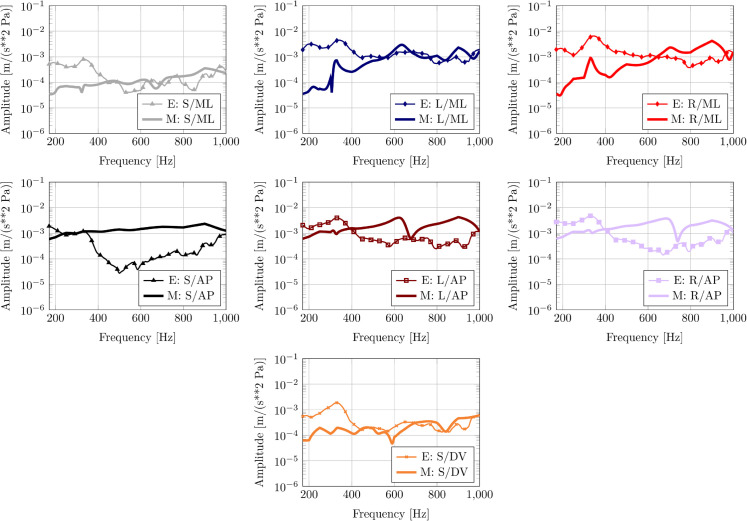
Experiment 13Sept2023 with the natural skull. Experiment FRF (E) compared with HVA FRF computed with model (M). Top row: Motion of skull and the left and right tympanic bone in the mediolateral (ML) direction. Middle row: Motion of skull and the left and right tympanic bone in the anteroposterior (AP) direction. Bottom row: Motion of skull in the dorsoventral (DV) direction.

Fig 5 shows the frequency response function (FRF) curves in the mediolateral direction for the experiment from 13Sept2023 on the natural skull. Furthermore, Fig 5 shows the response curves in the anteroposterior direction. The model curves clearly pick up on the natural frequencies that involve vibration of the tympanic bullae in the ML direction (near 320 Hz and between 600–650 Hz). A notable amplitude mismatch is observed for low frequencies, where the model under predicts the response to frequencies below 500 Hz when compared to the experiment. This discrepancy suggests that the acoustic energy delivered in the model at low frequencies was less than in the physical experiment. This issue is considered further in the discussion below. Finally, the dorsoventral vibration of the skull is shown in Fig 5. As for the other directions, the model underestimates the amplitude for low frequencies.

**Fig 6:**
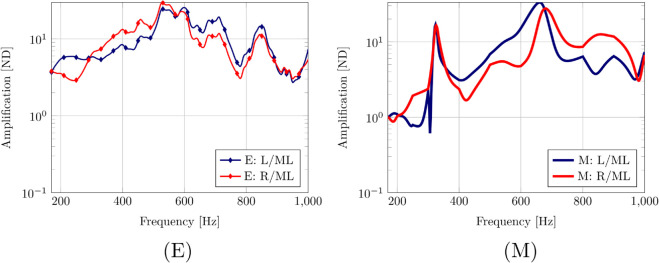
Experiment 13Sept2023 with the natural skull. Amplification of ML motion. Experiment FRF (E) compared with HVA FRF computed with model (M). Amplification of the mediolateral (ML) motion of the left and right tympanic bone relative to the skull.

Fig 6 presents the amplification of mediolateral accelerations of the tympanic bullae relative to the skull. While the peak amplification magnitude roughly agrees reasonable well with experimental observations, the amplifications for low frequencies are again underestimated. The similarity metric FRFSM can be applied to the pair of curves of amplification. Here we take the average of the amplification for the left and right bulla in the experiments and compare with the average of the amplification for the left and right bulla in the model: the result is 0.435 for the metric. The pronounced resonance near 330 Hz in the model curves matches the estimate of their natural frequencies, but those peaks are not detected in the experimental curves. An explanation of that may be that the amplification is a ratio of outputs, which may be large when the numerator is large or when the denominator is small, and in the experiment the mediolateral response of the skull (denominator) for the low frequencies is an order of magnitude larger in the experiment than in the model (see Fig 5).

## 4 Discussion

Overall, the model demonstrates strong performance in reproducing important biomechanical features of mysticete sound reception. The peak amplification magnitude predicted by the model agrees reasonably well with experimental observations. In particular, the model successfully captures the amplification of the motion of the tympanic bullae relative to the periotic bones. Similarity metrics also indicate that the model FRFs are fairly close to those observed experimentally. However, the agreement is not perfect, with the most prominent differences in their low-frequency behavior.

To better understand the sources of these discrepancies, we explore the sensitivity of the model to various input parameters and assumptions. This analysis provides insight into how material properties, boundary conditions, and loading assumptions influence model accuracy and can inform future refinement efforts.

### 4.1 Parametric study for the replica skull

The model results presented in Fig 4 were based on nominal (best-estimate) values for the input parameters. Here we consider the uncertainty of the input parameters for the plastic replica skull. Specifically, we performed a parametric study where input parameters were varied as shown in Table 6. A factorial design analysis was conducted with two factors, each considered at two levels, low and high [45]. (Each of the independently varied parameters is called a factor, and the change of the outputs associated with either the low or the high value is representative of the rate of change of the result with respect to the change in the factor.) The impact of these parameter variations was assessed using the quality of the match between the experiment and the model in terms of the FRFSM similarity measure (only the median, the confidence interval is not reported here).

**Table 6:** Ranges of input parameters for the plastic replica skull. E = Young’s modulus;eak rho = mass density.

Quantity	low	nominal	high
E [GPa]	1.885	2.095	2.305
rho [${kg/m}^{3}$]	907	1008	1109

**Fig 7:**
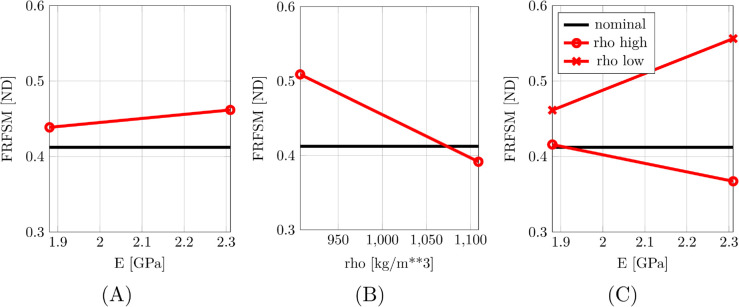
Main effects and interaction for the plastic replica skull. Main effects: (A) increasing the Young’s modulus moderately improves the similarity between the experimental FRFs and the model FRFs; (B) increasing the mass density deteriorates the similarity of the FRFs. Interaction: (C) There is a strong interaction between the two factors [45].

The main effects of the parametric study were found to be 0.023 for the Young’s modulus and -0.117 for the mass density, as illustrated in Fig 7. These values indicate that increasing the Young’s modulus moderately improves the similarity between the experimental and model FRFs, while increasing the mass density worsens the agreement. This is consistent with physical expectations: higher stiffness leads to elevated natural frequencies, bringing the model response closer to the experimentally observed resonances, whereas increased mass lowers the system’s resonant frequencies, reducing alignment with the experimental data. As expected, there is a strong interaction between the two factors, as together they jointly affect the natural frequencies of the system (the interaction manifests as different slopes of the lines that indicate the change in the similarity metric due to the change of the Young’s modulus as calculated for different values of the mass density factor); the model is improved when both Young’s modulus is increased and mass density is decreased.

### 4.2 Parametric study for the natural skull

The model results for the natural skull, shown in Fig 5, were based on nominal values of the input parameters, as for the replica skull. Here we consider the uncertainty of the input parameters for the natural skull. To assess the influence of parameter uncertainty on the model’s agreement with the experimental results, we conducted a parametric study focused on the parameters used for the natural skull configuration. Table 7 describes the ranges of the parameters considered. The experimental design is considered with two levels of each factor, low and high. The Young’s modulus and the mass density of both the skull and the TPC was varied, as were the loss factors of both the skull and the TPC. While the range of values investigated was approximately 20% for the Young’s moduli and mass densities, a broader range of values was explored for the loss factors, where there is a large amount of uncertainty present.

**Table 7:** Input parameters for the natural skull. Esk = Young’s modulus of the skull; Etp = Young’s modulus of the TPC; rhosk = mass density of the skull; rhotp = mass density of the TPC; lfs = loss factor of the skull; lft = loss factor of the TPC.

Quantity	low	nominal	high
Esk [GPa]	7.2	8.0	8.8
Etp [GPa]	31.5	35.0	38.5
rhosk [${kg/m}^{3}$]	990	1100	1210
rhotp [${kg/m}^{3}$]	2250	2500	2750
lfs [ND]	0.2	0.3	0.45
lft [ND1]	0.01	0.05	0.1

**Fig 8:**
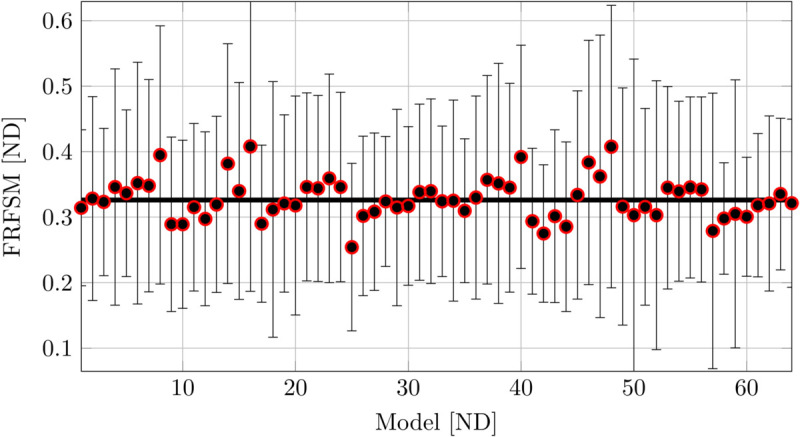
Parametric study with the natural skull. Experiment 13Sep2023 compared with HVA model. The model is varied by changing the factors, the Young’s modulus of the skull and of the TPC, the mass density of the skull and of the TPC, and the loss factor of the skull and of the TPC. The similarity for the nominal values is shown by a bold horizontal line. The results are shown as the median FRFSM across the seven sensors, and the confidence interval is presented as whiskers. For a two-level experimental design with six factors there were 64 different models (runs).

Fig 8 illustrates the sensitivity of the model - experiment agreement when the Young’s modulus, the mass density, and the loss factor of both the skull and the TPC were varied (six factors in total). The similarity of the frequency response curves shows some dependency on the factors, however the change is limited to a few percentage points compared to the nominal values.

Among the parameter combinations evaluated, models 8, 16, 40, and 48 in Fig 8 represent the “best” combinations of the factors in that they increase the similarity of the model and the experiment. These “best” models correspond to a low Young’s modulus of the TPC and high mass density of the TPC, combined with elevated values of the loss factors for both the skull and the TPC. The remaining factors appear to contribute little to the outcome. In summary, the variability (uncertainty) of the material input parameters has a limited effect on the match between the model and the experiment (at most ≈25% improvement of the FRFSM similarity metric to approximately 0.4).

### 4.3 Error of pressure-loading interpolation

The interpolation of the pressure loading along the frequency axis introduces an error. While this error could be completely eliminated by computing the distribution of the incident acoustic pressure on the surface of the bones for *each* of the 416 frequencies within the sweep, it would be exceedingly expensive, as discussed in Sect 2.2.1. For computational expediency, we use interpolation in frequency of snapshots of the pressure computed by the PRESS model at 100, 200, ..., 900, and 1000 Hz. Table 8 shows the similarity measures for the pressure loading being only interpolated from 100, 300, 500, 700, and 900 Hz. This should be compared with Table 5: the maximum deviation of a similarity measure is approximately 13%. We consider this difference sufficiently small, considering all other uncertainties.

**Table 8:** Comparison with experiments for the natural skull. Reduced sampling of the frequencies for the pressure loading calculation. Similarity measures between the FRFs at the seven virtual sensors obtained with HVA models using mesh 3 and the experiments. Similarity across the seven sensors reported with the median and the confidence interval in parentheses as ($\tilde{s}$, $2{\tilde{s}}^{mad}$).

Experiment date	CSSF2	CSAC	FRFSM
13Sept2023	(0.872, 0.141)	(0.249, 0.042)	(0.284, 0.118)

### 4.4 Uncertainties of the loading

It was realized at the outset of the experimental program that the ideal progressive plane-wave conditions of the incident acoustic wave could not be realized in the available pool of water (Navy TRANSDEC facility in San Diego [17]). The length of the required acoustic pulse for each frequency would allow standing waves to develop due to reflections from the pool’s boundaries, despite the “anechoic” design of the pool.

Indeed, the pressure loading experienced by the physical specimens was affected by the finite dimensions of the pool and the necessity for a steady-state pattern to develop, which dictated a relatively long pulse time. This allowed waves to bounce off the boundaries and create resonance (standing-wave) patterns. Contrariwise, the computational model assumed loading by an idealized train of progressive (as opposed to standing) plane waves in the water.

Clearly, there was a discrepancy between the loading of the experimental artifacts and of the finite element models. The experimental artifacts were subjected to a more complex pressure field than the one applied in the simulations, which likely contributed to discrepancies in the observed frequency response functions.

**Fig 9:**
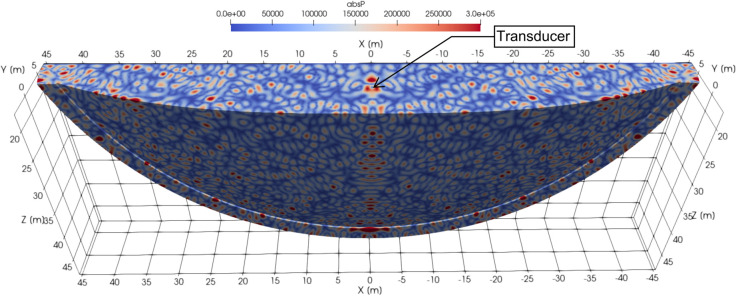
TRANSDEC pool: Steady state of the pressure field generated by a transducer at the center of the pool. 1000 Hz harmonic signal. Half of the pool is modelled. The bottom and sides of the pool are modelled as reflective, and the top of the pool is a pressure release surface.

Fig 9 illustrates the steady state acoustic pressure field (magnitude) for a 1000 Hz harmonic signal generated by a low-frequency transducer (obtained by [46]). The complex pattern of the standing waves are readily visible. During the experiment, the skull was located roughly one meter from the transducer, which was located at the center of the pool, at a depth roughly equal to half the depth of the pool. The wave pattern can be seen to display nodes spaced at intervals commensurate with the length of the skull, showing the potential for strong spatial variation in the incident loading.

Given that the experimental conditions were different from the assumptions employed in the computational models, we should not be surprised that differences in the results were found. This lead to the question How can we achieve a better match between the experimental conditions and the model conditions?

One possible aproach would be to model the total pressure field in the pool, including its interactions with the skull. This pressure field could then be applied as loading to the HVA model. However, this approach presents substantial challenges because of the difference in the length scales involved. The skull is roughly two orders of magnitude smaller than the pool itself, and the important anatomical details of the TPC are yet another two orders of magnitude smaller than the skull. Putting all these scales together into a single model would be challenging, requiring significant computational resources. Furthermore, the perturbation of this pressure field by the skull suspended in the water would have to be computed for a large number of frequencies. And still we could not be sure the experimental conditions would be closely matched: the impedance of the boundaries of the pool would need to be characterized, which is quite uncertain at present.

An alternative approach would be to repeat the experiments in an “infinite” body of water, for instance a lake of significant dimensions. In a way this would be the opposite of the previously suggested approach, as the experimental conditions would be brought closer to the model conditions.

In summary, the discrepancy between the idealized loading assumed in the model (plane harmonic wave) and the loading realized in the experiments (standing-waves of relatively complex form) likely contributed significantly to the observed mismatch between simulated and measured frequency response functions. How to control this confounding factor remains a challenge.

We need to point out that making predictions with the current model in situations where the loading by planar harmonic waves can be assumed with confidence, such as in an “infinite” body of water – the ocean – the modeling error can be expected to be considerably smaller. In these situations it would be controlled by the input uncertainties, and those were quantified above as having a limited impact on the agreement between the model and the experiment.

### 4.5 Validated or not validated?

Topics in this section are discussed in detail for instance in Trucano et al. [47].

It is important to note that in validation processes all sources of error need to be considered: Both errors introduced by the model and the errors of the experiments. In the experiments, we must consider the experimental error, which the Table 4 attempted to quantify for the plastic replica: the agreement of two sets of results obtained for two separate experiments led to FRFSM of approximately 0.8. In other words, just repeating the experiment twice produces results that differ by approximately 20%.

For the model, listed in the approximate order of magnitude, we find the arithmetic error, the discretization error, the input error, and the modeling (idealization) error. The first two errors, arithmetic and discretization, are controlled rather well. The error of the input parameters has been quantified by the sensitivity analyses above. So, it remains to quantify the modelling error (error introduced by the idealizations and assumptions that go into the model). For the natural skull, we found FRFSM of 0.326 (Table 5), which gives us an idea of the magnitude of the modelling error: given that we lose approximately 20% of the agreement between the model and the experiment due to the experimental error, the remainder of approximately 50% can be ascribed to the modeling error. (The reader will recall that the lack of agreement between the assumed loading and the loading realized in the experiments constituted the bulk of the modeling error.)

The current understanding of scientific validation activities is well captured by the statement “The process of determining the degree to which a computer model is an accurate representation of the real world from the perspective of the intended model applications” from [47]. The keywords critical in the present context are “intended model applications”. Thus, the similarity measures reported here as metrics indicate success of the validation process only as related to the intended model application: If the intent requires computer-model predictions that are of very high fidelity, let us say such that all similarity metrics >0.9 [48], the conclusions about the success of the present validation exercise will be very different from those reached if the intended application only requires attainment of similarity metrics >0.3.

Consider here as the intended application the prediction of the amplification of the motion of the tympanic bullae relative to the skull in the mediolateral direction. As described in Sect 3.4, the similarity metric FRFSM equal to 0.435 was calculated for the averages of the amplification for the left and right side. This value needs to be compared with a threshold above which we can claim successful validation. However, how to set a rational value for this threshold is at the moment up for debate. It is worth stating explicitly that validation is not binary; rather, it is a continuum of outcomes. So picking a threshold should really be approached in a Bayesian way as a tool for adjusting our confidence in the model.

What if the user of the described modeling pipeline wished to extend the studies to another mysticete? The use of a validated model in a class of similar problems is justified. However, the user could not claim that this validation meant the model could be applied to odontocetes. The mechanisms of hearing are likely different, and odontocetes would then be in a different class of problems. Extrapolation of a validated model to a class different from that for which the model was validated cannot be justified.

## 5 Conclusions

This paper describes an effort to validate computational (finite element) models of the skull bones of a young gray whale using experimental data obtained from both a natural skull and on a 3D-printed plastic replica. Model predictions were compared with experimental results using similarity metrics derived from frequency response curves of acceleration, with acoustic pressure in the water surrounding the skull serving as the excitation input. Overall, the agreement of the model and experiment was only moderate: the similarity metrics rarely reached the level of good match (0.7 or more).

A significant finding is that the model accurately reproduced the natural frequencies of the skull and the tympanic bullae, although not consistently in all directions and for all sensor locations. Importantly, the amplification of the tympanic bullae’s accelerations relative to the base of the skull was seen in the model curves, as in the experiments. While the overall shape of the frequency response curves from the model matched the experiments reasonably well, there are some significant discrepancies.

To investigate the source of these discrepancies, we performed a full factorial sensitivity analysis varying the Young’s modulus, density, and loss factors for both the skull and tympanoperiotic complex (TPC). Although some parameter combinations improved the match - favoring lower TPC stiffness, higher TPC density, and higher damping - these changes only improved the similarity by a few percent. Overall, material property uncertainty alone cannot explain the observed lack of fit.

We also considered the effect of loading conditions. There is mismatch between the idealized plane-wave loading used in the computational model and the complex standing-wave patterns realized in the pool experiments. This mismatch in loading conditions may largely explain the poor agreement in frequency response curves. In the finite pool, reflections from the walls and surface create resonant standing-wave pressure fields that deviate significantly from the progressive plane waves assumed in the simulation.

The value of the present validation effort is threefold: (i) It enables placing plausible bounds on estimates of the sound reception capability inherent in the build of the mysticete skull; (ii) It identifies the main source of the modeling error; and (iii) It suggests how the validation experiments could be improved in the future.

As validation is an ongoing process, the present effort represents a first step on the road towards attaining a reliable and high fidelity modeling capability, and this journey will be continued with refinements in both experimental design and computational modeling.
